# Suggestion of a Novel Classification Based on the Anatomical Region and Type of Bilateral Fatigue Femoral Fractures

**DOI:** 10.3390/medicina59091572

**Published:** 2023-08-29

**Authors:** Christos Koutserimpas, Dimitrios Kotzias, Efstathios Chronopoulos, Symeon Naoum, Konstantinos Raptis, Athanasios Karamitros, Konstantinos Dretakis, Maria Piagkou

**Affiliations:** 1 Department of Orthopaedics and Traumatology, “251” Hellenic Air Force General Hospital of Athens, 11525 Athens, Greece; dimit.kotzias3535@gmail.com (D.K.); naoumsimeon@gmail.com (S.N.); kraptis1981@hotmail.gr (K.R.); karameter@yahoo.gr (A.K.); 2 Department of Anatomy, School of Medicine, National and Kapodistrian University of Athens, 11527 Athens, Greece; mapian@med.uoa.gr; 3 2nd Department of Orthopaedics, “Hygeia” General Hospital of Athens, 15123 Athens, Greece; kostasdretakis@gmail.com; 4 Laboratory for Research of the Musculoskeletal System, School of Medicine, National and Kapodistrian University of Athens, 14561 Athens, Greece; stathi24@yahoo.gr

**Keywords:** femur fracture, fatigue injury, fracture anatomical site, stress fracture, classification

## Abstract

*Purpose*: Bilateral fatigue femoral fractures (BFFF) represent an extremely rare clinical entity. The present study introduces a novel classification, in order to categorize the BFFFs and provide a thorough review of all these, so far in the literature, reported cases. *Methods*: The BFFF were classified taking into account the anatomical region of the femoral fracture; (fh): femoral head, (sc): sub-capital, (pt): peri-trochanteric, (st): sub-trochanteric, (s): shaft, (d): distal femur and the fracture type (complete or incomplete); type I: bilateral incomplete fractures, type II: unilateral incomplete fracture, and type III: bilateral complete fractures. Type III was further subdivided into type IIIA: bilateral non-displaced fractures, type IIIB: unilateral displaced fracture, and type IIIC: bilateral displaced fractures. Furthermore, a meticulous review of the PubMed and MEDLINE databases was conducted to locate all articles reporting these injuries. *Results*: A total of 38 patients (86.8% males), with a mean age of 25.3 years, suffering BFFFs were identified from the literature search. The mean time interval from symptoms’ onset to diagnosis was 54 days. According to the proposed classification, 2.6% of the fractures were categorized as type I (h), 36.8% as type I (sc), 2.6% as type I(st/s), 7.9% as type I (s), 2.6% as type I (d), 5.4% as type II (fh), 26.3% as type II (sc), 2.6% as type IIIA (st), 2.6% as type IIIA (d), 5.4% as type IIIB (sc), 2.6% as type IIIB (d) and 2.6% as type IIIC (sc). Surgery was performed in 52.6%, while non-operative treatment was followed in 47.4% of the population. Regarding the fracture type, 75% of type I fractures were conservatively treated, while 91.7% and 66.6% of type II and III fractures were surgically treated. For patients treated conservatively, the mean time from diagnosis to return to previous status was 260 days, while for patients treated surgically, 343 days. *Conclusions*: BFFFs, although rare, may pose a diagnostic and therapeutic challenge. The present classification offers valuable information and may act as a guide for the management of these patients.

## 1. Introduction

Stress fractures represent a relatively common clinical entity and are frequently encountered in emergency departments. Stress fractures can be divided into fatigue and insufficiency fractures [1,2]. A fatigue fracture is a focal failure of normal bone, caused by repetitive applied stress, while an insufficiency fracture is a focal failure of abnormally weakened bone [1,3].

Fatigue fractures located the lower extremity are prevalent injuries among individuals engaged in endurance and high load-bearing activities like running, military training, and aerobic exercises [1,2]. Fatigue fractures’, located in the lower extremity, incidence is estimated to be between 0.7% and 20% of the total injuries seen in sports medicine. It is of note that runners experience fatigue fracture rates of approximately 16% of all their injuries. Regarding the anatomical distribution of lower extremities, the most frequent fatigue fractures manifest in the tibia (23.6%), but they also manifest in other areas, including the tarsal navicular (17.6%), metatarsals (16.2%), femur (5–13%), and pelvis (1.6%) [2,3,4]. These stress fractures result from overuse and/or excessive strain, occurring when the pace of stress-induced microfractures surpasses the bone’s repair capacity [2].

Regarding the femur, along with atypical femoral and pathological fractures, stress fractures comprise atraumatic or minimally traumatic injuries, caused by relatively low-energy mechanism that typically would not be expected to lead to a fracture [1]. Clinical manifestation, imaging findings and treatment options of atraumatic fractures often overlap and may misdirect, leading to delayed diagnosis or misdiagnosis and consequently may result in suboptimal management [1]. Understanding the underlying etiology and pathophysiology of each fracture type is of paramount importance for prompt diagnosis and successful treatment. Femoral stress fractures commonly manifest with discomfort in various areas such as the hip, groin, gluteal, thigh, or knee, contingent on their location. Athletes might also experience indistinct thigh pain coupled with generalized tenderness, particularly in cases of femoral neck stress fractures [2,5]. The pronounced morbidity associated with femoral stress fractures arises from substantial compression and tensile forces exceeding the body’s weight. This results in a morbidity rate spanning from 20% to 86%, as documented in literature, encompassing complications like complete fractures, malunion, impingement, nonunion, avascular necrosis, and arthritic changes. The femoral shaft represents the most prevalent site for stress fractures, trailed by the lesser trochanter and intertrochanteric region [1,2,3,4]. Regardless of the specific region, athletes consistently report pain during physical activity, which may be replicated during passive range of motion, particularly when performing internal rotation or hopping on the affected limb. Detecting femoral stress fractures has proven challenging, often resulting in an average diagnostic delay of approximately 14 weeks. Conventional X-rays usually yield normal results, reaffirming that MRI remains the premier diagnostic tool for visualizing femoral stress fractures [1,2,3].

The femur represents a relatively common anatomical region of fatigue fractures, accounting for approximately 11% of all these injuries [4]. However, simultaneous fatigue fractures of both femurs represent an extremely rare clinical entity, that is mostly observed in military recruits and less commonly in athletes and ordinary active individuals [5,6,7]. Due to their low incidence, these fractures are often mis-or-under-diagnosed [1].

The purpose of the present study is to introduce a novel classification, in order to categorize bilateral fatigue femoral fractures (BFFFs) and provide a thorough review of all reported in the literature such cases.

## 2. Methods

The novel classification of the BFFF was based on the anatomical region of the fracture and the type of fracture (complete or incomplete) and associated the classification with the final treatment. Based on the anatomical region of the femur, the fracture is described as (fh): femoral head, (sc): sub-capital, (pt): peri-trochanteric, (st): sub-trochanteric, (s): shaft and (d): distal femur, while based on the fracture type, the fracture is described as type I: bilateral incomplete femoral fractures, type II: unilateral incomplete femoral fracture, type III: bilateral complete femoral fractures. Type III is consequently divided into 3 subtypes, type IIIA: bilateral non-displaced femoral fractures, type IIIB: unilateral displaced femoral fracture, and type IIIC: bilateral displaced femoral fractures. Furthermore, in type II if the fracture sites are not in the same femoral region, the incomplete fracture is written first, while the symbol (*) is used in case the complete fracture was displaced. Furthermore, in type IIIB, the displaced fracture site is written first (in case the two fractures are located in different anatomical regions) (Table 1).

### 2.1. Search Strategy 

A meticulous online search of the PubMed and MEDLINE databases was conducted, to locate articles reporting BFFFs, free of time frame limitation for the present study. Two combined terms, with the word “bilateral” included at least once, were used for the literature search: “femoral stress fracture”, “femoral neck stress fracture”, “bilateral”, “bilateral stress fracture”, “femoral stress fracture”, “subtrochanteric stress”, “trochanteric stress”. Following the studies’ identification, individual references listed in each included publication were further investigated for the ascertainment of additional cases. 

### 2.2. Selection Criteria

Studies reporting prospective or retrospective clinical and radiological data, involving BFFFs were included. Studies not published in the English language or did not present adequate data about the patient’s demographics (gender and age), the type of fracture (complete or incomplete) or the final treatment were not included in the study. Expert opinions, book chapters, or in-vitro investigations, as well as abstracts in scientific meetings, were also excluded.

### 2.3. Data Extraction 

Titles and abstracts of studies were retrieved using the search strategy and extracted independently by two different authors (DK & CK), who screened the titles and abstracts of the retrieved papers for eligibility and analyzed the full-text articles that met the eligibility criteria. 

The examined variables were: 1. demographic characteristics of the patient (gender and age), 2. physical activity of the patient, 3. time-interval from pain development to diagnosis, 4. diagnostic imaging tools, 5. fracture type based on radiological data available and categorized according to the proposed classification, 6. final treatment and 7. time-interval from diagnosis to return to previous status.

Data were recorded and analyzed using Microsoft Excel 2019 (Microsoft Corporation, Redmond, WA, USA).

## 3. Results

**Figure 1:**
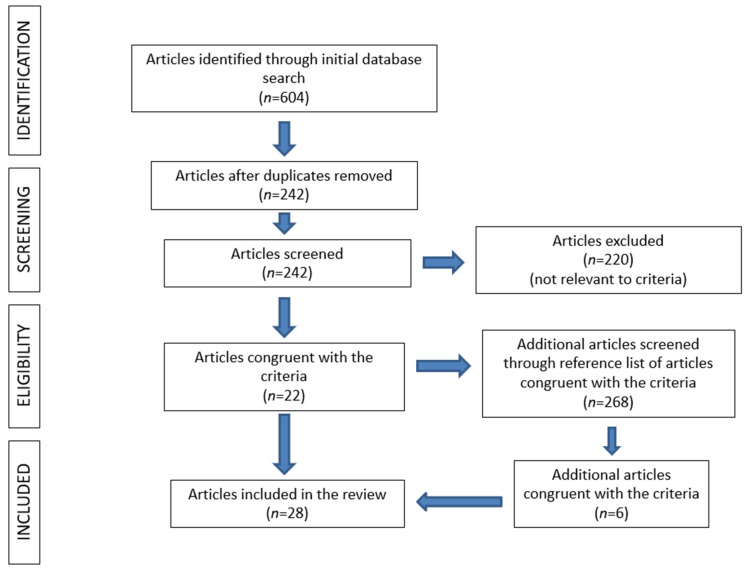
Search strategy.

A total of 242 articles were identified and finally, 22 of them (9.1%) met the inclusion criteria [6,8,9,10,11,12,13,14,15,16,17,18,19,20,21,22,23,24,25,26,27,28]. Additionally, 6 studies were retrieved after careful investigation of the references’ lists of the pertinent articles [5,7,29,30,31,32]. The search strategy is exhibited in Figure 1. In total, 38 patients with bilateral fatigue femoral fractures were included, extracted from 28 studies [5,6,7,8,9,10,11,12,13,14,15,16,17,18,19,20,21,22,23,24,25,26,27,28,29,30,31,32].

### 3.1. Patients Characteristics

Regarding the demographic characteristics, the studied population’s mean age was 25.3 [standard deviation (SD) = 10.8], while 33 (86.8%) were males and 5 (13.2%) females. Adequate information concerning the patients’ physical activity was reported in 27 (96.4%) studies [5,6,7,8,9,10,11,12,13,14,15,16,17,18,19,21,22,23,24,25,26,27,28,29,30,31,32]. A total of 23 patients (60.5%) were engaged in military training, 10 (26.3%) in sports or increased intensity physical exercise, while 4 (10.5%) reported no particularly increased physical activity, except from their regular occupation. All patients included were healthy, with no history of underlying disorder that could compromise bone quality.

### 3.2. Diagnostic Process

Time interval from initial pain development to final diagnosis was reported for 33 patients (86.8%) and ranged from 2 to 365 days with mean time being 54 days [SD = 68.3] [6,7,8,9,10,11,12,13,14,15,16,17,18,19,20,21,22,23,24,25,26,27,28,29,30,31,32]. 

Regarding the imaging modalities used during the diagnostic process, X-rays were performed in 37 (97.4%) patients, magnetic resonance imaging (MRI) in 20 (52.6%) patients, bone scintigraphy scan (BS) in 16 (42.1%) and computerized tomography (CT) scan in 2 (5.3%). Definite diagnosis of bilateral fatigue femoral fractures was established with MRI in 17 patients (44.7%), with BS in 11 (28.9%), with X-rays in 8 (21.1%) and with CT in 2 (5.3%).

### 3.3. Bilateral Fatigue Fractures of the Femur According to the Proposed Classification

Based on the type of the femoral fracture (Type I, Type II, Type III), 20 (52.6%) patients were diagnosed with a bilateral incomplete femoral fracture or Type I, 12 (31.6%) were diagnosed with a unilateral incomplete femoral fracture or Type II and 6 (15.8%) were diagnosed with a bilateral complete femoral fracture or type III. More particularly, out of the 6 Type III fractures, 2 (33.3%) were categorized as type IIIA, 3 (50%) as type IIIB and 1 (16.7%) as type IIIC. Regarding the type II fractures, 4 (33.3%) out of the 12 complete fractures were displaced (Type II*). 

Regarding the anatomical region of the femoral fracture, 3 (7.9%) fractures were located at the (fh), 27 (71.1%) at the (sc) region, 1 (2.6%) at the (st) region, 3 (7.9%) at the (s) site, 3 (7.9%) at the (d), while no fracture was reported at the (pt) area. In 1 (2.6%) patient, bilateral incomplete fractures were not located at the same femoral region. One fracture was located at the sub-trochanteric area, while the contralateral at the shaft of the femur (st/s) [32]. 

Therefore, according to the proposed classification, 1 (2.6%) out of the 38 fractures was categorized as type I (h), 14 (36.8%) as type I (sc), 1 (2.6%) as type I (st/s), 3 (7.9%) as type I (s), 1 (2.6%) as type I (d), 2 (5.4%) as type II (fh), 10 (26.3%) as type II (sc), 1 (2.6%) as type IIIA (st), 1 (2.6%) as ype IIIA (d), 2 (5.4%) as ype IIIB (sc), 1 (2.6%) as type IIIB (d) and 1 (2.6%) as type IIIC (sc) [Table 2].

### 3.4. Final Treatment in Association with the Fracture Type

The final management of bilateral fatigue femoral fractures was reported for all 38 patients included. A total of 18 (47.4%) patients were treated conservatively, while surgery, unilateral or bilateral, was performed in 20 (52.6%) patients. Regarding the fracture type, 15 (75%) out of 20 Type I fractures were treated conservatively, compared to 5 (25%) that were managed surgically (*p*-value = 0.03), 1 (8.3%) out of 12 Type II fractures was treated conservatively and 11 (91.7%) surgically (*p*-value = 0.004), while 2 (33.3%) out of 6 Type III fractures were treated conservatively and 4 (66.7%) surgically (*p*-value = 0.4).

Conservative treatment included initially bed rest and consequently gradual weight-bearing ambulation until full activity levels, while surgical treatment included various surgical procedures based on the femoral region of the fracture site. Out of the 20 patients treated surgically, 2 (10%) suffered from bilateral (h) fractures, 17 (85%) from bilateral (sc) fractures and 1 (5%) from bilateral (d) fractures. Of the 2 (h) fractures, 1 (50%) was treated unilaterally with ORIF and contralaterally with total hip arthroplasty and 1 (50%) bilaterally with fibular strut allograft. Of the 17 (sc) fractures, 16 (94.1%) were treated bilaterally with ORIF and 1 (5.9%) unilaterally with ORIF and contralaterally with total hip arthroplasty. Finally, one bilateral (d) fracture was treated with intramedullary nail at both sides.

### 3.5. Time-Period from Diagnosis to Return to Previous Status

Time-interval between the definite diagnosis and the return to previous status of activity was reported for 32 (84.2%) patients and the mean time-interval was found to be 299 days [SD = 280.8]. More precisely, for patients treated conservatively the mean time-period from diagnosis to return to previous levels of activity was 260 days [SD = 287], while for patients treated surgically the mean time was 343 days [SD = 276]. 

## 4. Discussion

Fatigue femoral fractures, represent a bone injury related to repetitive stress and overload on the femur. Unlike acute fractures that result from a single, high-energy, traumatic event, fatigue fractures develop gradually over time, as a result of repetitive loading and inadequate time for bone remodeling and repair. There are various contributing factors to the occurrence of fatigue fractures, that can be divided into extrinsic and intrinsic [4]. Extrinsic factors include training regimen, footwear, training surface and type of sport, while intrinsic factors are defined as characteristics of the individual person, such as gender, age, overall fitness status, as well as skeletal, muscle, joint and biomechanical factors [4]. Active females are reported to have a higher incidence of fatigue fractures compared with active males [4].

The present study introduced a novel classification of BFFFs, based on the anatomical region of the injury and the fracture type (complete/incomplete). Furthermore, a meticulous review of all such reported cases was conducted and these cases were categorized according to the proposed classification.

The femur is the longest and strongest bone in the human anatomy, responsible for supporting human body weight and facilitating various movements. However, when subjected to excessive or repetitive stress, such as in activities involving running, jumping, or prolonged standing, the bone may experience micro-damage that exceeds its ability to repair itself. In the present study, the majority of patients (60.5%) were engaged in military training, followed by those (26.3%) engaging in sports or increased intensity physical exercise.

The symptoms of fatigue femoral fractures may vary but typically include pain, tenderness, and swelling in the thigh or groin area, while since there is no traumatic event, these injuries may be misdiagnosed. In relevance to the anatomic region, differential diagnosis may differ. For instance, in the femoral shaft, without evidence of a traumatic event, adductor or rectus femoris muscle strains may be included in the initial differential diagnosis, while in the femoral neck, trochanteric bursitis should also be excluded. It is of note that the mean time-interval between symptoms’ onset and definite diagnosis was almost 2 months (54 days) in the present study. Regarding imaging techniques establishing the final diagnosis, MRI was utilized in most patients (44.7%), followed by BS (28.9%). Advanced imaging modalities may be necessary in these injuries, since these fractures may not appear on initial X-ray views. MRI’s capacity to reveal soft tissue and bone edema, makes this imaging modality the gold-standard for diagnosis fatigue fractures. An early indicator of stress fractures is the presence of bony edema, a feature not easily discernible through standard radiographic methods [1,2,4]. Radiographs may complement clinical history by showcasing details like periosteal bone formation, cortical margins, and fracture lines, which may not be observable within the initial 2 weeks of experiencing symptoms [4]. However, radiographs are insufficient for identifying acute stress fractures, as it might take around 3 weeks for cortical irregularities and periosteal reactions to become apparent. Consequently, alternative imaging approaches are suggested. While CT scans have proven useful in stress fracture diagnosis, they lack the sensitivity of MRIs in simultaneously assessing soft tissue. Bone scans (scintigraphy) are highly sensitive for detecting stress fractures, yet their usage is limited due to concerns about radiation exposure, while the emergence of MRI sensitivity has diminished their utilization. Although the available literature on ultrasonography is restricted, it holds promise for future applications [1,2,4].

The increased prevalence of physical activity in modern society, coupled with advancements in imaging techniques, has contributed to notable increase in the documented occurrence of stress fractures over recent decades. These fractures now constitute more than 10% of cases encountered in a typical sports medicine practice. Bilateral fatigue fractures had not been classified. We proposed a novel classification facilitating prompt communication between clinicians, since these injuries may appear more often. All such fractures reported in the literature could be classified according to the proposed classification. Furthermore, it seems that regarding the anatomical region of bilateral fatigue femoral fractures, most of them occur at the sub-capital site (71.1%), while most patients suffer from incomplete fractures bilaterally (52.6%; Type I). 

Regarding treatment, the decision between conservative and operative management is mainly based on the anatomical region of the fracture and the fracture’s type (complete/ incomplete). This information is provided from the proposed classification. More particularly, it seems that the majority of type I fractures (incomplete bilaterally) are treated non-operatively (75%), while most complete fractures (type II and III) are managed surgically (91.7% of type II and 66.7% of Type III). In association with the anatomical region of the fracture, most fractures treated surgically were located at sub-capital femoral region (17 cases; 44.7%). In terms of surgical treatment of a femoral fracture, the anatomic location is of utmost importance in dictating the most suitable intervention [33]. Intracapsular hip fractures consist of subcapital and transcervical ones and they may be treated with pinning (for younger patients) and hemiarthroplasty or total hip arthroplasty (for older patients) [33,34]. Extracapsular hip fractures occur distal to the attachment of the joint capsule to the femur and can be divided into basicervical, intertrochanteric and pertrochanteric subgroups, with osteosynthesis usually being achieved with an intramedullary nail. Femoral shaft and subcondylar fractures should be reduced and fixated either with intramedullary nails or plates [33,34].

This study has some limitations. It is of note that BFFFs represent a relatively rare injury, while treatment of femoral fractures is indicated from the anatomic location and the patient’s age. However, in the recent years there has been an increase in individuals starting recreational running and in those taking up ultra-endurance running [35,36,37]. Hence, it is assumed that there will be an increase of fatigue injuries in the near future, making such a classification more relevant. Furthermore, the present study represents the first effort to classify the BFFFs providing information regarding the anatomical site and the type of fracture. This information may be useful for clinicians to decide upon further management of these patients. Moreover, the classification could be useful for research purposes, as well as communication between clinicians

## 5. Conclusions

Fatigue femoral fractures are a significant concern for active individuals, particularly those involved in high-impact activities. The BFFFs, although rare, may pose a diagnostic and therapeutic challenge. The present classification offers valuable information that may guide management of these patients. Understanding the causes, symptoms, and appropriate management of these fractures is crucial for promoting recovery, preventing complications, and optimizing long-term bone health.

## Figures and Tables

**Table 1:** The novel classification of bilateral femoral fatigue fractures, taking into account the anatomical site and the pattern the fracture. In Type II if the fracture sites are not in the same femoral region, the incomplete fracture is written first, while the symbol (*) is used in case that, the complete fracture is displaced. Furthermore, in Type IIIB, the displaced fracture site is written first (in case that the two fractures are in different anatomical regions).

Novel Classification of Bilateral Femoral Fatigue Fractures			
Fracture Type		Anatomical Region of the Fx Site	
Type I	Bilateral Fx incomplete	fh	Femoral head
Type II ² (*)	Unilateral Fx incomplete	sc	Sub-capital
Type III	Bilateral Fx complete	pt	Peri-trochanteric
Type IIIA	Bilateral Fx non displaced	st	Sub-trochanteric
Type IIIB	Unilateral Fx displaced	s	Shaft
Type IIIC	Bilateral Fx displaced	d	Distal femur

Fx: Fracture; ² If the Fx is not in the same region, the incomplete is written first; *: displaced Fx.

**Table 2:** Main characteristics and classification of the reported bilateral fatigue femoral fractures. In type II fractures, the symbol (*) is used in case the complete fracture was displaced.

Demographics	Gender [Male (M)/Female (F)]	Age/ Mean Age (Years)	Physical Activity	Time Interval from Pain Development to Diagnosis [(Days (d)]	Fracture Type Based on Koutserimpas Classification	Final Treatment	Time Period from Diagnosis to Return to Previous Status
Rengman E (1960) [30]	M	21	Military training	90 d	II (sc)	Non-operative	365 d
D J Blatz (1981) [8]	F	15	Track running	14 d	I (s)	Non-operative	28 d
Vento JA et al. (1986) [25]	M	53	Jogging	14 d	II (sc/sc *)	Operative	-
Fullerton LR Jr et al. (1988) [5]	M	29	Military training	-	I (sc)	Non-operative	730 d
Fullerton LR Jr et al. (1988) [5]	M	30	Military training	-	I (sc)	Non-operative	730 d
Fullerton LR Jr et al. (1988) [5]	M	18	Military training	-	I (sc)	Non-operative	42 d
Fullerton LR Jr et al. (1988) [5]	M	33	Military training	-	I (sc)	Non-operative	730 d
Fullerton LR Jr et al. (1988) [5]	M	18	Military training	-	I (sc)	Non-operative	42 d
Stoneham MD et al. (1991) [32]	M	23	Military training	49 d	I (st/s)	Non-operative	-
Toren A et al. (1997) [24]	M	5	Roller blade	10 d	I (s)	Non-operative	35 d
Song WS et al. (2004) [31]	M	22	Military training	90 d	II (fh)	Operative	-
Song WS et al. (2004) [31]	M	21	Military training	60 d	I (fh)	Non-operative	180 d
Weind KL et al. (2005) [7]	F	15	Cross country running	42 d	I (s)	Non-operative	98 d
Salminen ST et al. (2007) [22]	M	19	Military training	18 d	IIIA (d)	Non-operative	730 d
Ross K et al. (2008) [21]	M	14	Cross country running	28 d	I (d)	Non-operative	30 d
Hutchinson PH et al. (2008) [10]	M	15	Lacrosse training	60 d	IIIB (d)	Non-operative/ Operative	270 d
Naranje S et al. (2012) [18]	Μ	34	Military training	2 d	IIIB (sc)	Operative	365 d
Yoon PW et al. (2012) [28]	M	27	Military training	21 d	II (fh)	Non-operative/ Operative	730 d
Muzaffar N et al. (2013) [16]	M	37	Dambali dancing	180 d	IIIA (st)	Non-operative	365 d
Nemoto O et al. (2013) [29]	M	24	Military training	12 d	II (sc)	Operative	730 d
Khadabadi NA et al. (2015) [13]	M	25	Work related	30 d	I (sc)	Operative	540 d
Webber BJ et al. (2015) [26]	M	23	Military training	5 d	I (sc)	Operative	120 d
Lee GW et al. (2016) [14]	M	10	Taekwondo training	28 d	I (sc)	Non-operative	70 d
Moo IH et al. (2016) [15]	M	19	Military training	7 d	I (sc)	Non-operative/Operative	120 d
Moo IH et al. (2016) [15]	M	18	Military training	7 d	II (sc/sc *)	Non-operative/Operative	120 d
Moo IH et al. (2016) [15]	M	20	Military training	14 d	II (sc)	Operative	120 d
Oliveira US et al. (2016) [6]	M	43	Work related	365 d	I (sc)	Operative	-
Santoso A et al. (2017) [23]	M	37	Work related	21 d	I (sc)	Non-operative	60 d
Kanwat H et al. (2019) [12]	F	50	Work related	120 d	II (sc)	Operative	365 d
Jalan D et al. (2020) [11]	M	36	Running	90 d	II (sc/sc *)	Operative	730 d
Rajkumar N et al. (2020) [20]	F	38	-	30 d	IIIB (sc)	Operative	-
Yoon HK et al. (2020)	M	20	Military training	60 d	I (sc)	Non-operative	70 d
Yoon HK et al. (2021) [27]	M	19	Military training	60 d	II (sc)	Non-operative/Operative	70 d
Yoon HK et al. (2021) [27]	M	20	Military training	3 d	II (sc/sc *)	Operative	70 d
Yoon HK et al. (2021) [27]	M	18	Military training	60 d	II (sc)	Non-operative/Operative	70 d
Nam DC et al. (2021) [17]	F	43	Trampoline exercise	60 d	I (sc)	Non-operative/Operative	-
Pongsamakthai W et al. (2021) [19]	M	20	Military training	90 d	IIIC (sc)	Operative	730 d
Heig T et al. (2022) [9]	M	29	Military training	42 d	I (sc)	Non-operative	120 d
	Male: 33 (86.8%) Female: 5 (13.2%)	Mean age: 25.3		Mean time-interval: 54 d	I (fh): 1 (2.6%) I (sc): 14 (36.8%) I (st/s): 1 (2.6%) I (s): 3 (7.9%) I (d): 1 (2.6%) II (fh): 2 (5.4%) II (sc): 10 (26.3%) IIIA (st): 1 (2.6%) IIIA (d): 1 (2.6%) IIIB (sc): 2 (5.4%) IIIB (d): 1 (2.6%) IIIC (sc): 1 (2.6%)	Non-operative: 18 (47.4%) Operative bilateral: 13 (34.2%) Operative unilateral: 7 (18.4%)	Mean time-period: 299 d

## Data Availability

Not applicable.
